# Spin Polarization Properties of Pentagonal PdSe_2_ Induced by 3D Transition-Metal Doping: First-Principles Calculations

**DOI:** 10.3390/ma11112339

**Published:** 2018-11-21

**Authors:** Xiuwen Zhao, Bin Qiu, Guichao Hu, Weiwei Yue, Junfeng Ren, Xiaobo Yuan

**Affiliations:** 1 School of Physics and Electronics, Shandong Normal University, Jinan 250014, China; zhaoxiuwen@stu.sdnu.edu.cn (X.Z.); qiubin@stu.sdnu.edu.cn (B.Q.); hgc@sdnu.edu.cn (G.H.); physics_yue@163.com (W.Y.); 2 Institute of Materials and Clean Energy, Shandong Normal University, Jinan 250014, China

**Keywords:** spin polarization, transition metal doping, PdSe_2_, first-principles calculations

## Abstract

The electronic structure and spin polarization properties of pentagonal structure PdSe_2_ doped with transition metal atoms are studied through first- principles calculations. The theoretical investigations show that the band gap of the PdSe_2_ monolayer decreases after introducing Cr, Mn, Fe and Co dopants. The projected densities of states show that p-d orbital couplings between the transition metal atoms and PdSe_2_ generate new spin nondegenerate states near the Fermi level which make the system spin polarized. The calculated magnetic moments, spin density distributions and charge transfer of the systems suggest that the spin polarization in Cr-doped PdSe_2_ will be the biggest. Our work shows that the properties of PdSe_2_ can be modified by doping transition metal atoms, which provides opportunity for the applications of PdSe_2_ in electronics and spintronics.

## 1. Introduction

The successful stripping of graphene has greatly stimulated people’s interest in the research of two-dimensional (2D) materials, and it has been widely used in the design of electronic devices due to its remarkable physical and chemical properties [1,2,3,4,5]. However, the zero band gap of graphene limits its application in electronics, which leads to the emergence of other 2D materials with a hexagonal structure beyond graphene, such as black phosphorous, boron nitride and transition metal di-chalcogenides (TMDCs) [6,7,8,9,10,11,12,13]. Two-dimensional hexagonal structure materials are widely used in various aspects, especially in spintronics. The long spin-coherence lengths and high spin-orbit coupling offer more opportunities for the fabrication of 2D spintronic devices, such as graphene nanoribbon electrodes, graphene spin valve, MoS_2_ switching of spin currents and so on [14,15]. Researching and inducing magnetic structures of 2D materials are vital but challenging topics in 2D spintronic devices. Various strategies to obtain magnetism have been performed, such as the introduction of defects [16,17], electric field and strain modulation [18,19], 3D transition-metal (TM) atom doping [20,21], surface adsorption [22,23,24], doping combined with adsorption [25] and so on.

With the tremendous efforts in research, more novel 2D materials came out. For example, a new class of layered material formed by noble metals (e.g., Pd and Pt) with S or Se atoms has been extensively investigated in recent years [26,27,28]. In fact, the hexagonal structure is the dominant motif in the ocean of 2D material. However, there is still a lack of experimental studies on the pentagonal structure. Recently, novel 2D PdSe_2_ composed of a pentagonal structure has been successfully exfoliated by Oyedele, et al., which provides exciting opportunities for the research of pentagonal 2D materials [29]. PdSe_2_ is a new material, it has strong-interlayer coupling [30,31], in addition, the air-stability and anisotropy of PdSe_2_ have been proved, moreover, few-layer PdSe_2_ behaves ambipolar semiconducting, with high electron-apparent field-effect mobility. Furthermore, the monolayer PdSe_2_ has an indirect band gap of about 1.3 eV, while the band gap of bulk PdSe_2_ is about 0 eV, hence the band gap of PdSe_2_ can be tuned between 0–1.3 eV, this is different from that in MoS_2_ with a value between 1.2–1.8 eV [28].

Compared with previously realized isotropic planar structures, the unique atomic configuration, coupled with the buckling structure, result in exotic mechanical properties, with an unusual negative Poisson’s ratio and ultrahigh mechanical strength. The spin-orbit coupling is strong, and a topological quantum phase transition also can be tuned in PdSe_2_. Therefore, the PdSe_2_ is a favorable candidate for designing novel 2D spintronic devices [29,32]. However, pristine PdSe_2_ is nonmagnetic, which will hinder its usage in spintronic devices. Recently, the half-metallic ferromagnetism of PdSe_2_ monolayer with hole-doping under uniaxial stress has been investigated by Zhang, et al. [33], which shows that Stoner ferromagnetism can be induced through hole-doping. In this paper, we studied the magnetic properties of PdSe_2_ with 3D TM atom dopants (Cr, Mn, Fe, Co and Ni atoms) by means of first-principles calculations.

## 2. Theoretical Model and Computational Details

In our first-principles calculations, we adopt the Vienna Ab Initio Simulation Package (VASP) equipped with the projector-augmented-wave (PAW) method to study the electron-ion interactions [34]. The Perdew-Burke-Ernzerh (PBE) functional of the generalized gradient approximation (GGA) is considered to treat the electron exchange correlation, which produces the correct ground-state structure of the combined systems [35,36,37]. In our calculations, the model of the 2 × 2 × 1 pristine single-layer PdSe_2_ (24 atoms) is given, the distance between adjacent PdSe_2_ layer set as 20 Å to avoid the effects induced by periodic boundary conditions. The Brillouin zone sampling uses a 11 × 11 × 1 Monkhorst-Pack grid. To reach a convergence of the total energy, the cut-off energy with 400 eV has been adopted in each calculation. The convergence threshold of the residual forces on each atom is 0.01 eV/Å and the total energy changes are less than 10^−4^ eV. The layers of PdSe_2_ are mainly held together by van der Waals forces [29,38], therefore, van der Waals (vdW) is introduced in the density functional method. 

## 3. Results and Discussions

**Figure 1:**
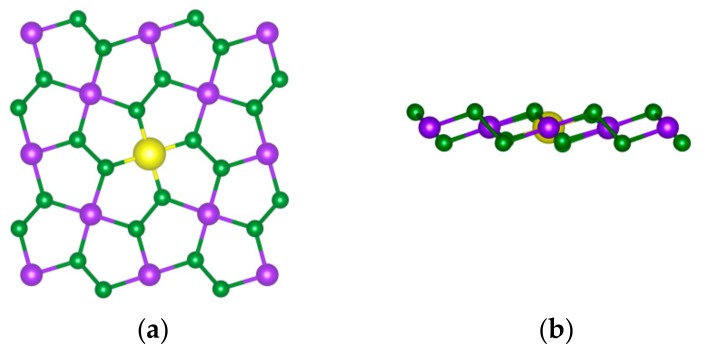
Top (**a**) and side (**b**) views of PdSe_2_ configuration. Green, purple and yellow balls represent Se, Pd, and dopant (Cr, Mn, Fe, Co and Ni atoms) respectively.

**Figure 2:**
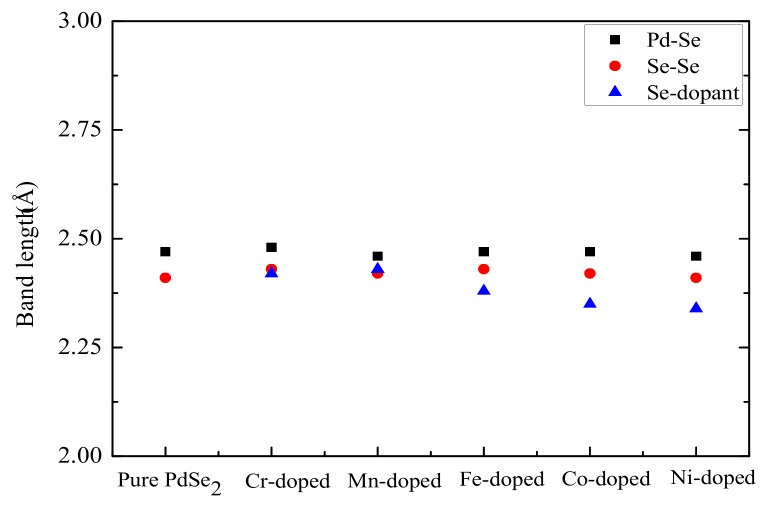
Bond length for different PdSe_2_ systems.

The fully relaxed structures are shown in Figure 1. Through comparison with the primitive cell, it can be found that the configuration of the doped system has no significant change. The changes of the bond lengths in PdSe_2_ before and after doping are given in Figure 2. It can be observed that the lengths of Pd-Se and Se-Se remain unchanged while the lengths of Se-dopants changed, but the changes are very subtle. These results suggest that the 3D TM atom doping has little effect on the bond lengths of PdSe_2_, hence, it is advisable to embed TM atoms at the dopant sites.

The spin polarization energy (E_pol_), which is defined as the energy difference between the nonmagnetic and ferromagnetic states (E_pol_ = E_non_ − E_fer_), is shown in Table 1. The positive E_pol_ means that the system favors ferromagnetism, in this case the energy of the ferromagnetic state is lower than that of the nonmagnetic state. Our calculated results are shown in Table 1, in which one finds that the system favors ferromagnetism under Cr, Mn, Fe and Co doping, however, the system with Ni dopants favors nonmagnetism due to the negative E_pol_.

**Figure 3:**
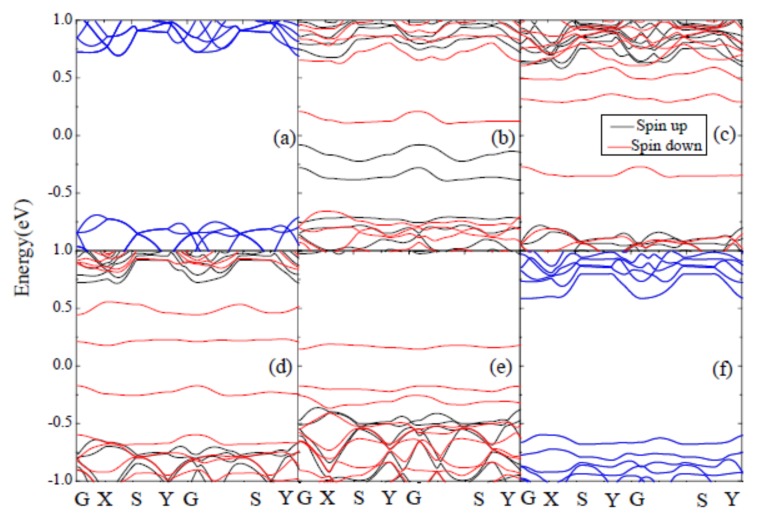
Electronic band structures of different doped systems, (**a**) pristine system, (**b**) Cr-doped system, (**c**) Mn-doped system, (**d**) Fe-doped system, (**e**) Co-doped system, (**f**) Ni-doped system, respectively.

The electronic band structures for different doped systems are depicted in Figure 3. It can be observed that the pristine PdSe_2_ monolayer is a semiconductor with a band gap of about 1.37 eV, which is consistent with previous experimental and theoretical results [28,29,30], however, significant changes happen after introducing the Cr, Mn, Fe and Co dopants. The band gap decreases for all systems, the value of band gaps is 1.30, 1.31, 1.32, 1.30 and 1.17 eV respectively, and there are new electronic states in the band gap. It also can be seen in Figure 3 that the band structure of pristine PdSe_2_ is degenerate, there is no spin polarization. For Cr-, Mn-, Fe- and Co-doped PdSe_2_, the band structures are nondegenerate, they show spin polarization. However, the Ni atom has little contribution to the magnetism of PdSe_2_. In order to make further comparisons, the magnetic moment for different doped systems are also calculated based on the Bader analysis [39], which is shown in Table 1. It can be found that the Cr-doped system has the biggest magnetic moment, about 3.71 μB, which means that the Cr doping can induce the strongest ferromagnetic coupling.

**Figure 4:**
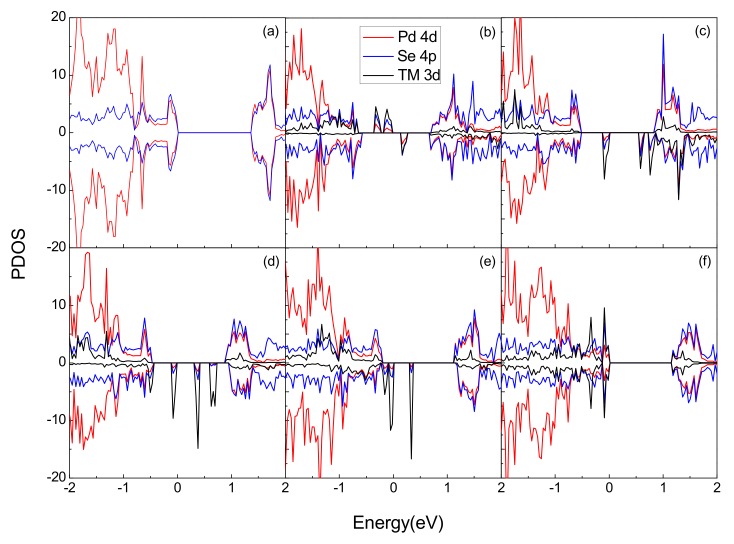
Projected density of states (PDOS) of the different doped systems, (**a**) pristine system, (**b**) Cr-doped system, (**c**) Mn-doped system, (**d**) Fe-doped system, (**e**) Co-doped system, (**f**) Ni-doped system, respectively.

The electronic states near the Fermi level (E_F_) have great influence on the electron structure. Thus, it is of great importance to investigate the spin polarization properties near the E_F_. In Figure 4, the electronic properties for different systems are depicted by displaying the projected density of states (PDOS) with spin-up and spin-down states. The d-states of the Pd atom and p-states of the Se atom contribute most to the electronic states of pristine PdSe_2_ in both our calculation and previous work [28]. In fact, pristine PdSe_2_ is non spin polarized, while after doping the Cr, Mn, Fe and Co atoms, obvious spin asymmetry can be observed near the E_F_. The p-d orbital couplings between the TM atom and PdSe_2_ lead to the generation of the new spin states. The Cr doping system has the biggest spin-splitting, which corresponds to the strongest ferromagnetism, this result is consistent with the magnetic moment calculated in Table 1. The new electronic states for the Cr, Mn, Fe and Co doped systems near the E_F_ are mainly originated from the 3D orbits of TM atoms. Moreover, the charge transferred from TM metals to PdSe_2_ will fill these new spin nondegenerate states, which lead to the systems being spin polarized. Nevertheless, there is no spin split for the Ni doping system, which means that one Ni atom doping is incapable of inducing ferromagnetism in PdSe_2_. Furthermore, through comparing the curves of Figure 4b–e we can find similar characteristics, this shows that the doping mechanism of the four systems are similar. 

**Figure 5:**
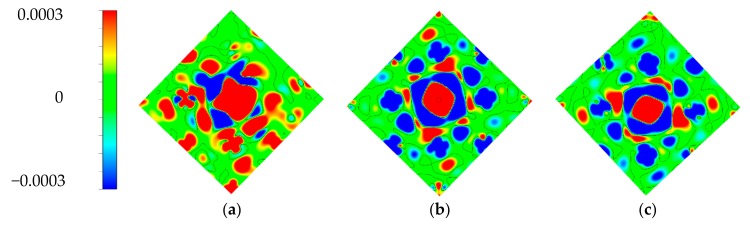

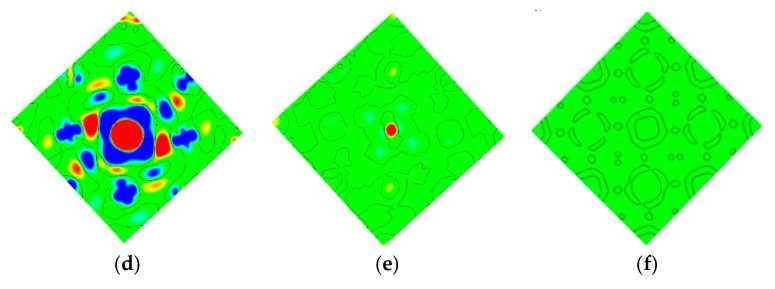
Spin density distributions for different doped systems. (**a**–**f**) correspond to Cr-, Mn-, Fe-, Co-, Ni- doped and pristine PdSe_2_, respectively. The 2D planes are determined by two Pd atoms and the dopants for the doped system, three Pd atoms for pristine PdSe_2_.

The spin density distributions of the TM doped and pristine PdSe_2_ systems are also given in Figure 5. The spin density is defined as Δ*ρ*_s_ = *ρ*_↓_ − *ρ*_↓_, where *ρ*_↑_ represents the spin-up charge density, *ρ*_↓_ is the spin-down charge density, the red and blue region in Figure 5 correspond to Δ*ρ*_s_ > 0 and Δ*ρ*_s_ < 0, respectively. It can be seen from Figure 5a–d that the system is spin polarized. Through comparing the patterns, it can be found that the area of spin density gradually decreases for the Cr-, Mn-, Fe- and Co-doped systems, however, there is almost no spin distribution for the Ni-doped and pristine system. This phenomenon is consistent with the above discussions of the PDOS and magnetic moments. Furthermore, the magnetism of PdSe_2_ induced by the Cr, Mn, Fe, Co and Ni atoms is gradually reduced, this is because the ability of the above TM atoms to lose electronics gradually weakens, then the amount of charge transfer gradually decreases. 

To support the above results, we further analyzed the charge transfer ΔQ from the TM atoms to the PdSe_2_ monolayer of the four systems with the Bader analysis [39], the values of ΔQ are shown in Table 1. It is clear that the Cr-doped system has the biggest ΔQ, about 0.82 e, and the value gradually decreases for the Mn-, Fe-, Co- and Ni-doped systems, they are 0.75, 0.54, 0.37 and 0.25 e, respectively. This is because different TM atoms have different abilities to lose electrons, therefore, they have different effects on the PdSe_2_. We can go further and say that coulomb interactions between the different transferred charges and PdSe_2_ make the electronic structure change differently, hence, the varying degrees of spin polarization appear in the PdSe_2_ monolayer. 

## 4. Conclusions

The electronic structure and spin polarization properties of pentagonal the PdSe_2_ monolayer with TM (Cr, Mn, Fe, Co and Ni) atom doping have been studied through the density functional theory. By calculating the spin polarization energy and the magnetic moment, one can see that the PdSe_2_ systems show different spin polarization properties and Cr-doped PdSe_2_ has the most stable and strongest magnetism, while the Ni doping cannot induce magnetism in PdSe_2_. In terms of electronic band structure, the band gap decreased after the Cr, Mn, Fe, Co and Ni atom doping, the value of band gaps is 1.30, 1.31, 1.32, 1.30 and 1.17 eV, respectively, and the branches of spin up and down is nondegenerate for the Cr, Mn, Fe, Co doped systems. The electronic properties for different systems are discussed through the PDOS, the new spin states originated from the p-d orbital couplings between the TM atoms and PdSe_2_. In addition, the spin density distributions and charge transfer for different PdSe_2_ systems also prove that the TM atom doping can induce magnetism in PdSe_2_ and the biggest spin polarization occurred in the Cr-doped system. Our calculations can contribute to the studies of spin polarization in pentagonal PdSe_2_, and the promising prospect of PdSe_2_ in spintronic applications.

## Figures and Tables

**Table 1:** Calculated spin polarization energy, charge transfer and magnetic moments of PdSe_2_ with different dopants.

System	Cr-Doped	Mn-Doped	Fe-Doped	Co-Doped	Ni-Doped	Pristine PdSe_2_
E_pol_ (eV)	2.78	1.61	0.82	0.21	−1.25	-
ΔQ (e)	0.82	0.75	0.54	0.37	0.25	-
Magnetic Moment (μB)	3.71	2.99	1.99	1.00	0.00	0.00
